# The Inside Out of Lentiviral Vectors

**DOI:** 10.3390/v3020132

**Published:** 2011-02-14

**Authors:** Stéphanie Durand, Andrea Cimarelli

**Affiliations:** 1 Department of Human Virology, Ecole Normale Supérieure de Lyon, 46 Allée d’Italie, 69364 Lyon, France; E-Mail: steffdurand@gmail.com; 2 INSERM U758, 46 Allée d’Italie, 69364 Lyon, France; 3 University of Lyon 1, 50 Avenue Tony Garnier, 69364 Lyon, France; 4 IFR128 BioSciences Lyon-Gerland, Lyon-Biopole, 69364 Lyon, France

**Keywords:** lentivirus, lentiviral vector, gene therapy, HIV, SIV, EIAV, FIV

## Abstract

Lentiviruses induce a wide variety of pathologies in different animal species. A common feature of the replicative cycle of these viruses is their ability to target non-dividing cells, a property that constitutes an extremely attractive asset in gene therapy. In this review, we shall describe the main basic aspects of the virology of lentiviruses that were exploited to obtain efficient gene transfer vectors. In addition, we shall discuss some of the hurdles that oppose the efficient genetic modification mediated by lentiviral vectors and the strategies that are being developed to circumvent them.

## 1. Introduction: On Lentiviruses and Their Natural Cellular Targets

### 1.1. Virus Replication and Pathology

Lentiviruses owe their *lenti* appellative (slow in *latin*) to the long period of time elapsing between the initial infection and the onset of the disease, that can protract over a period of months or even years. Viruses belonging to the *Lentivirus* genus are present in primates, ungulates (horse, cattle, sheep and goat) and felids (cat) (for reviews see [1,2]). Primates are the natural host for several lineages of closely related simian and human immunodeficiency viruses (SIV and HIV, respectively) that are the etiologic agents of the acquired immunodeficiency syndrome (AIDS) [3–5]. In monkeys, SIVs are divided in five lineages according to their host species: sooty mangabey, SM; african green monkey, AGM; chimpanzee, CPZ; mandrill, MND; syke, SYK. Transmission of these simian viruses to humans gave raise to two genetically distinct viruses: HIV-1, closely related to SIV_CPZ_ and HIV-2, closely related to SIV_SM_. Both HIV-1 and HIV-2 cause AIDS. However, while HIV-1 induces a rapid syndrome in the absence of anti viral treatment and is responsible for AIDS worldwide, HIV-2 infected individuals develop AIDS with substantially slower kinetics and its distribution is essentially restricted to West Africa [6–8]. Interestingly, SIVs are capable of developing a peaceful relationship with their host, since SIV infection is non-pathogenic in the natural host. However, the infection becomes pathogenic when transmitted to a different species, as is the case in experimental infections of monkeys, or as has been the case in humans (a number of recent reviews illustrate these aspects in detail, [9–13]. Among ungulates, sheep is the natural host for the Visna/maedi virus (VMV), goats for the caprine arthritis-encephalitis virus (CAEV), cattle for the bovine immunodeficiency virus (BIV), and horses for the equine infectious anemia virus (EIAV) [2]. Finally, domestic and wild cats are infected with the feline immunodeficiency virus (FIV) that induces an AIDS-like syndrome [14].

Historically, the first description of a lentiviral induced disease came from the observation of a slowly progressive disorder in the sheep flocks present in Iceland during the 1950s. This disease was a severe form of pneumo-encephalopathy that gave its name at its causal virus (paralysis and wasting, *i.e.*, *visna* and labored breathing, known as *maedi*, in Icelandic) [15,16]. Despite the variety of pathologies they induce, lentiviral infections share several common features. *De novo* infection is characterized by an acute phase of viral replication that is transitory and that rapidly progresses into a chronic period. This long chronic phase in which viral replication is substantially diminished characterizes the pathogenesis induced by most lentiviruses, from the severe immunodeficiency caused by primate and feline immunodeficiency viruses, to the synovitis in CAEV-infected goats or to the severe pneumo-encephalopathy observed in VMV-infected sheep. During this period, lentiviruses continue to replicate and gradually subvert, as is the case of primate immunodeficiency viruses, host defenses. After this chronic phase the disease becomes manifest. Not all lentiviruses are associated to a disease, as is the case for BIV which causes only mild symptoms in infected cattle (namely lymphocytosis), although more pathogenic strains of BIV have recently been isolated [17,18]. Besides a certain number of exceptions to this general description do exist. For example, lentiviral infection can rapidly lead to the onset of the disease, as observed in HIV-1-infected newborns, or in CAEV-infected kid goats. In the case of EIAV infection, the chronic phase is not established after the initial infection, but rather after the disease. Indeed, infected animals develop anemia quite rapidly after infection and subsequently enter a relatively asymptomatic chronic state. This state is interrupted by cycles of peak viremia and disease that protracts during the entire life span of the animal [2].

### 1.2. Myeloid Cells as a Preferential Target for Lentiviruses

Myeloid cells include a large panel of cell types with specialized functions. Blood monocytes are precursors that leave the circulation in response to tissue damage or infection and enter tissues where they differentiate into macrophages or dendritic cells (DCs). These are professional antigen presenting cells (APCs) that play a central role in the orchestration of host immune responses [19–22]. Following a similar migratory pattern, monocytes are also able to cross the blood-brain barrier before differentiating into microglia cells in the central nervous system. Overall, these cell types are not homogeneous, but are constituted by highly heterogeneous cell subtypes performing specific functions. For example, at least two sets of monocytes co-exist in the blood: CD16^−^CD14^+^ cells, the most abundant monocyte population in the blood and CD16^+^CD14^+^ cells, a minor monocyte population with a more activated phenotype (about 1 to 5% of circulating monocytes) [23–26]. The presence of distinct cell subtypes is common not only to monocytes, but also macrophages and DCs. To add to the extreme complexity of cells of the myeloid lineage, these cells have the ability to polarize in response to the surrounding environment (for example into M1 or M2 macrophages) and to exert distinct functions [22]. A more extended introduction on the plasticity of myeloid cells would fall beyond the scope of this review and the reader is referred to other reviews that have covered these aspects [22,26,27].

Despite the fact that lentiviruses infect different cell types, all seem to share an exquisite ability to target cells of the myeloid lineage *in vivo* (as for VMV, CAEV, HIV, SIV and FIV) [28–35]. In the case of EIAV the tropism for monocyte-macrophages is extreme, because these cells seem to be the only ones infected (including Kupffer cells that are macrophage-like cells in the liver) [36]. In the case of HIV, the virus is primarily transmitted as a CCR5-tropic virus, a co-receptor present on cells of the myeloid lineage in addition to gut-associated lymphocytes [37–40]. To strengthen this point, it is interesting to note that lentiviral infection causes a variety of neurological disorders that result from viral replication in the central nervous system (CNS) [41–43]. Given that monocytes are the sole cell type that under normal conditions is able to traverse the blood-brain barrier, it is clear that the virus must use these cells to gain access to this privileged site.

The relationship established between myeloid cells and lentiviruses is multifaceted. For example, EIAV and VMV integrate as proviruses into circulating monocytes, however their genome is not expressed until these cells differentiate into macrophages, a phenomenon called post-integration silencing [44–48]. Instead, HIV-1 delays integration for days after entry in non-stimulated monocytes, thus achieving pre-integrative silencing [49–53]. Albeit different, these silencing strategies may allow the virus to remain under cover within monocytes thus minimizing their exposure to the immune system. Afterward when monocytes enter the tissues and become activated, the virus is able to exit this covert phase and resumes its replication cycle [44,52,54]. This mode of action offers a double advantage to the virus: to profit from the migratory behavior of monocytes to reach sites in which viral spread can occur, as the lymph nodes or the CNS; to be present within the very cells that ought to instruct antiviral responses. The consequence of the latter is the possibility for the virus to modify the behavior of these antigen presenting cells and thus to influence antiviral immune responses.

These considerations are not meant to diminish the importance that the infection of other cell types has in the pathogenesis caused by the different lentiviruses, but is meant to suggest that these viruses may have evolved certain common features that allow them to target myeloid cells. In light of their multiple roles, these cells are of interest for a wide variety of applications that range from anti-cancer strategies, to vaccination and antiviral immunity [55,56]. From a virological point of view, these cells are non-dividing cells and this implies that the viral genome must traverse an intact nuclear membrane to access the cellular genome. Among retroviruses, lentiviruses have evolved the most efficient machinery to achieve this goal, a property that is highly valuable in gene therapy.

## 2. Basic Aspects of Virology Applied to Lentivectors

### 2.1. An Introduction to the Early Phases of the Viral Life Cycle

The viral life cycle can be divided into two main phases: one in which the viral genome is transferred into the host cell and one in which this genome is expressed and viral propagation assured. The main interest of viral vectors for gene therapy purposes lays in the first, collectively referred to as the early steps of the viral life cycle. This process can be defined as gene transduction (in gene therapy) or single cycle infection (in virology) and both terms will be used here. In the case of retroviruses, the infection starts with the engagement of a specific cellular receptor by the viral envelope and culminates in the integration of the neo-synthesized full length viral DNA into the host cell genome. The cellular receptor-viral Env engagement triggers the fusion between the plasma cell and the particle membranes and results in the release of a viral nucleoprotein complex (VNC) composed of the viral genome associated to viral and cellular proteins in the cytoplasm of target cells. The exact composition of VNCs has proven particularly difficult to establish due to different reasons. These structures are labile and change over time as they accommodate the conversion of the viral genome from an RNA to a double stranded DNA molecule. In addition, for reasons that are currently unknown, a large proportion of VNCs present during infection is non-functional and this complicates the analysis of the composition of truly infectious VNCs. Nonetheless, a number of notions as to their composition and trafficking have been gathered and will be discussed below. If this rather general description applies to all retroviruses, the infection of non-dividing cells requires the viral genome to traverse the nuclear membrane to access the host genome. This step, key for gene therapy purposes in non-dividing cells, is termed nuclear import.

### 2.2. An Efficient Nuclear Import, the Distinctive Feature of Lentiviruses

The nuclear membrane constantly regulates exchanges between the cytoplasm and the nucleus, apart from a brief period in which it is removed during mitosis. These exchanges depend on the nuclear pore, a multiprotein structure that allows free exchanges of small molecules, but that selectively controls the transport of molecules of a molecular weight superior to 40 kDa [57]. Proteins localized in the nucleus possess a nuclear localization signal (NLS) by virtue of which they are recognized by karyopherins, or importins, that are the cellular cytoplasm-nucleus transporters. In humans, karyopherins are divided in two families comprising multiple members (6 and 20 for karyopherins α and β, respectively). The NLS is recognized either directly by karyopherin β or via the karyopherin α adaptor that associates to karyopherin β. The cargo-karyopherin complex is transported through the nuclear pore into the nucleus via interactions with several of its constituents, the nucleoporins. The asymmetric distribution of the GTP/GDP bound forms of the Ras-related nuclear protein, the Ran GTPase, is responsible for the directionality of this transport toward the nucleus. The GTP-bound form of Ran (RanGTP) is primarily found in the nucleus, where it binds to the imported complex dissociating the karyopherin from its cargo. The karyopherin-RanGTP complex shuttles back into the cytoplasm, where it dissociates upon hydrolysis of GTP to GDP. RanGDP is then transported back to the nucleus by a specific transporter, the nuclear transport factor 2 (NTF2), so that the cycle can start anew. These movements are possible because of the high and low affinities that Ran displays for karyopherins in its GTP- or GDP-bound state, respectively. The directionality of the transport relies also on a Ran gradient that is maintained by the Ran guanine exchange factor (RanGEF, that charges Ran with GTP in the nucleus) and by the Ran GTPase activating protein (RanGAP, that mediates the hydrolysis of GTP in GDP in the cytoplasm).

For the purposes of viral infection, the nuclear membrane is an additional obstacle that can be bypassed either by taking advantage of its natural removal during cell division, or by traversing the nuclear pores. Intuitively, the latter is the only possibility available in non-dividing cells. The relationship between retroviruses and nuclear membrane remains surrounded by a number of questions. For example, although *simple* retroviruses are impaired in the transduction of growth-arrested cells, there is no proof that they truly access the nucleus in that short window of time during which the nuclear membrane is absent in dividing cells. Similarly, although lentiviruses must traverse the nuclear pore during the infection of non-dividing cells, it remains unclear whether this step is optional or obligatory during the infection of dividing cells.

Aside from these considerations and despite the fact that other retroviruses are able to accomplish infection of non-dividing cells [58–61], lentiviruses certainly appear the most efficient ones to carry out nuclear import. In light of their exquisite tropism for non-dividing myeloid cells, it is tempting to speculate that they have perfected this property through evolution.

Over the years, a number of studies attempted to identify determinants of nuclear import and, as most of the work has been focused on HIV-1, we shall use this virus as a paradigm for other lentiviruses. The literature on the subject is vast, and has often been contradictory due to the heterogeneity of the experimental systems used and to the relative complexity of nuclear import (for reviews, see [57,62–64]). However, a certain consensus is now apparent, as we will discuss below.

Historically, potential factors that mediate nuclear import were sought among components of VNCs. Among them, the search was directed at nucleophilic elements specific to lentiviruses. A number of proteins met these criteria, namely Matrix (MA), Integrase (IN) and Vpr. The first two are structural components of viral particles and are conserved among retroviruses. However, NLSs seem present uniquely in lentiviral MA and IN proteins. Instead, Vpr is a non-structural viral protein incorporated into particles and is coded solely by primate lentiviruses. Despite the fact that these proteins localized to the nucleus, their role during nuclear import turned out to be negligible, as most defects observed upon their mutagenesis were due to pleiotropic effects, rather than to an effect on nuclear import *per se* (compare the above mentioned reviews with older reviews on the subject, [65–67]. Retrospectively, failure to reveal a major role in nuclear import for these proteins was just as possible as the contrary would have been. Indeed, even if a protein is nuclear when expressed outside its context, it may not be so when part of a higher order molecular complex (such as the VNC). This may be due to different reasons, given that a particular protein may be present in limited amounts in the complex to drive it into the nucleus, or else it may not be exposed to physically associate to components of the nucleo-cytoplasmic transport machinery. Following a similar rationale, early studies focused on a particular sequence present almost exclusively in the genome of lentiviruses, named the central polypurine tract-central termination sequence (cPPT-CTS) [68]. The cPPT sequence is present roughly in the middle of the viral genome and, similar to the polypurine tract present at the 3′ of the viral genome, it resists RNaseH-mediated degradation and acts as an internal primer for viral DNA synthesis. The CTS is located downstream of the cPPT and is the site at which plus strand viral DNA synthesis terminates. Presence of the cPPT-CTS determines a discontinuity in the plus strand of completed viral DNA that yields a triple helix structure, a DNA flap, that is later resolved in an integrated provirus. Although a central discontinuity region has been evidenced in the genome of primate foamy viruses [69] and in yeast retrotransposons [70], it is unclear whether this region yields to the formation of a DNA flap and whether it acts similarly to the one present in lentiviruses [71]. In lentiviral vectors, presence of the cPPT-CTS exerts a positive effect on viral infectivity on most cells [72–74]. Although initial studies conferred a prime role to this structure in nuclear import [74], the first generation of lentiviral vectors was devoid of cPPT-CTS and was still capable of transducing non-dividing cells [75]. This and other studies tempered the enthusiasm over the role of the cPPT-CTS in nuclear import, implying that although it could contribute to the process, it was not its major determinant [76–79]. More recent data indicate a simpler role for the cPPT-CTS in promoting faster kinetics of reverse transcription [80]. This hypothesis is captivating, as lentiviral infection occurs in cells of low metabolism in which this process can be extremely long (such as myeloid cells). Thus, it is intriguing to speculate that this sequence may have evolved specifically in lentiviruses to maximize the speed at which reverse transcription is completed. In turn, fast completion of viral DNA synthesis may exert a protective effect on the viral genome for example by promoting its faster nuclear import, as suggested in [77,78], by contributing to structural rearrangements of VNCs or, more generally, by protecting it from cytoplasmic sensors that recognize RNA or single stranded DNA. In this respect, the cPPT-CTS has been recently shown to contribute to the protection of HIV-1 from members of the apolipoprotein B editing catalytic polypeptide 3 family (APOBEC3s), cytidine deaminases with marked antiviral activity [81–83]. These proteins are incorporated into virion particles where they deaminate single stranded viral DNA intermediates. Although APOBEC3s incorporation is countered by the viral protein Vif, residual molecules may escape Vif, especially in cells in which APOBEC3 members are expressed at high levels. In this case, the cPPT-CTS element minimizes the time of exposure of single stranded DNA, thus providing an additional level of protection against APOBEC3 proteins [81]. More generally, we do believe that the cPPT-CTS may exert a protective effect beyond APOBEC3s, because the advantage of lentiviral vectors bearing this sequence is manifest also in the absence of APOBEC3 proteins. We believe it likely that by promoting faster kinetics of reverse transcription, the cPPT-CTS may indirectly protect the viral genome from multiple attacks brought by deaminases, but also by other endonucleases and yet unidentified cellular factors.

If the above-mentioned viral elements contribute marginally to nuclear import, what are its true viral determinants? An important difference between the composition of VNCs obtained after lentiviral and gammaretroviral infection is their content in Capsid protein (CA). CA is the main structural component of viral cores within viral particles, but as soon as the virus accesses the cytoplasm of target cells, CA is progressively shed from VNCs. In the case of lentiviruses which are more apt for nuclear import than gammaretroviruses, this loss is more pronounced [84–88]. Is this difference important for nuclear import? A role for CA in nuclear import is suggested by several findings: the existence of specific CA mutants that behave differently in cycling *versus* non-dividing cells, as well as the nuclear import defect of HIV chimeric viruses bearing gammaretroviral CA [89–93]. At present, a number of questions surround the role of CA in nuclear import. In particular, if CA associates with specific cellular transporters to reach the nucleus, why would loss of CA from viral cores be beneficial for this transport? On the other hand, if shedding of CA from the VNCs uncovers other signals that mediate nuclear entry, which is the nature of these signals given that most of the components of VNCs have been found to play only a marginal role in this process? Does lentiviral CA promote a peculiar VNC trafficking through which VNCs gain preferential access to the nucleus? More generally, is nuclear import governed by a single key determinant which we have not yet found by multiple elements playing minor roles, or is it to be considered an overall promiscuous mechanism, as recently suggested in [94]?

Despite these questions, the difference in the extent of CA shedding from VNCs between lentiviruses and gammaretroviruses is compelling and suggests that VNCs reorganization may play a central role in nuclear import [92,95,96]. If this is true, a number of factors that affect the viral core stability and composition may indirectly influence nuclear import. One such factor may be reverse transcription itself that drives important structural changes in VNCs, or a structure such as the DNA flap, which may signal the end of the process [95,97]. From a strictly theoretical point of view, the possibility that the end of reverse transcription drives structural changes that enable the nuclear import of VNCs is intriguing, as this may be a mechanism to favor the entry of completed viral DNA molecules into the nucleus [98].

The above-mentioned studies focused on viral proteins. However, several studies identified cellular components of the nucleo-cytoplasmic transport machinery as capable of modulating the nuclear import of HIV-1: Nup85, Nup107, Nup133, Nup153, Nup155, Nup160, RanBP2, Transportin-SR2/TNPO3, importin alpha3 and Importin7 [99–103]. A direct interaction between these factors and viral proteins has been evidenced only in the case of Transportin-SR2/TNPO3 and Importin7 that associate with IN [104,105]. Although multiple evidence indicates that these two cellular proteins are involved in the nuclear import of HIV-1, the importance of IN during this step remains uncertain [106], as nuclear import occurs also in the absence of IN [78]. For the other cellular factors it remains unclear whether the role played in nuclear import is direct or indirect. In light of the functions of these proteins in a key cellular process (nucleo-cytoplasmic transport), this latter possibility cannot be excluded.

### 2.3. The Conversion of a Lenti-Virus into a Lenti-Vector

The concept of viral-based tools for gene delivery emerged for the first time in the early 1980s with vectors based on the Moloney Murine Leukaemia Virus (Mo-MLV) [107]. Vectors rely on the physical separation into different plasmids of proteins required for viral particle formation and infectivity (the packaging and the envelope constructs) and of *cis*-acting sequences sufficient to mobilize the viral genome (the transfer vector). The latter constitutes the core of the vector; a mini-viral genome devoid of viral open reading frames (ORFs), but carrying an expression cassette for the transgene of interest. As a consequence of the deletion of viral ORFs from the transfer vector, virions can undergo a single round of infection at the conclusion of which proviral DNA expresses only the transgene of interest. Among lentiviruses, HIV-1-based vectors were the first to be developed and gain wide usage for both fundamental and applied purposes, so that our description shall mostly focus on them again as a paradigm for other lentiviral-derived vectors.

**Figure 1.**
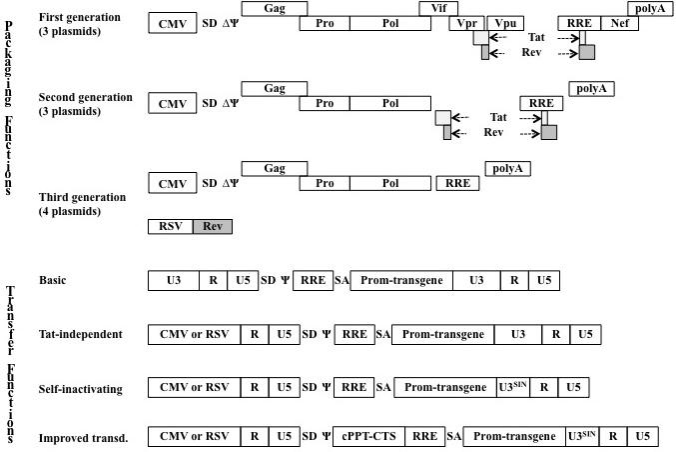
Evolution of lentiviral vectors based on HIV-1. The scheme simplifies schematically the evolution of packaging and transfer vectors. In the case of transfer vectors, only three major evolutions have been depicted, despite the existence of a number of modifications that still today continue to ameliorate the vectors performance. LTR, long terminal repeat; SA and SD, splice acceptor and donor; RRE, Rev-responsive element. Rev associates to the RRE on viral genomic RNA and allows its export from the nucleus and its efficient incorporation into virion particles [182].

The first gene delivery systems used replication-incompetent HIV-1 vectors to study different aspects of the viral life cycle in the early 1990s [108–113], but the key breakthrough came with the construction of vectors that, in contrast to MLV-derived ones, were capable of transducing non-dividing neurons when injected into rat brains [75]. The first vector generation was made of three plasmids in which the packaging functions were provided by an Env-coding plasmid and by a packaging plasmid expressing all viral ORFs except Env under the control of a CMV promoter (in which of course the packaging sequence had been removed). The transfer vector was composed of an expression cassette framed by two wild type long terminal repeats (LTRs) and bearing sequences required for viral RNA export in producing cells (the Rev-Responsive Element, RRE), genome packaging and reverse transcription (Figure 1 and Table 1). In the second generation packaging vectors, most accessory genes were eliminated (*vif*, *vpr*, *vpu* and *nef)* and only Tat and Rev were retained [114], while in the third, Tat was also removed and Rev was provided on a fourth plasmid [115] (third generation vectors are based on four plasmids instead of three). In the case of transfer vectors, a number of modifications contributed to increase the performance of gene transfer, as for example the use of post transcriptional regulatory elements that enhance the transgene transcriptional expression as the human hepatitis virus post transcriptional element (HPRE) [116,117], or the use of heterologous polyadenylation enhancer elements, as those derived from simian virus 40 (SV40) or β-globin [118,119], or the use of different internal promoters to express a particular gene (or gene products, as shRNAs) of interest.

However, three major modifications have shaped the evolution of transfer vectors from their initial version [75]. The first is the substitution of the 5′ U3 viral promoter for a heterologous promoter to allow the Tat-independent transcription of the transfer vector [115,118,120,121]. The second is a deletion of the enhancer/promoter sequence of the 3′ U3 [115,118,120,121]. By the gymnastic of reverse transcription, this deleted 3′ U3 sequence is copied at both ends of proviral DNA resulting in a provirus that lacks a functional U3 viral promoter. This deletion, at the basis of self-inactivating (SIN) vectors, increases the safety of lentiviral vectors due to the lack of expression of ψ-bearing mRNAs in transduced cells and to the minimization of gene activation in the proximity of the provirus integration site [122]. The third is the inclusion of the cPPT-CTS sequence that exerts a positive effect on transduction efficiency, despite controversies over its exact function [72,73,77,123].

Overall, the development of lentiviral vectors, that we have simplified here schematically, seeks to optimize the efficacy of gene transfer, while eliminating the potential dangers due to the use of retroviral vectors. Examples of this constant optimization have been the development of third and fourth generation vectors that minimize considerably the risks of generation of replication-competent recombinants (RCR), or the introduction of the SIN mutation that diminishes both the probability of RCR formation and impairs the promiscuous enhancer activity of the viral promoter.

## 3. Additional Aspects of the Biology of Lentiviral Vectors for Particular Applications

The notions outlined above have been at the basis of the development of lentiviral vectors. However, further efforts are ongoing to improve gene transfer, as for example those directed towards the targeting of specific cell types or the increase of the overall efficiency of transduction. These efforts aim at achieving the highest percentage of modified cells with the lowest viral input. Below, we will describe some of these ongoing efforts.

### 3.1. Pseudotyping of Lentiviral Vectors

Retroviral particles have an extraordinary ability to accommodate heterologous envelope proteins, and are referred to as pseudotyping, can acquire novel cellular tropism and intracellular behavior (for a review see [124]). Pseudotyping is common to all retroviruses, so that we will mostly outline here a few cases that are exclusive to lentiviruses in the transduction of quiescent and differentiated cells.

The first property specified by envelope proteins is the type of cell recognized by the viral particle. In this respect, if pantropic envelopes, such as the vesicular stomatitis virus G protein (VSVg), are used to mediate viral entry into a wide variety of cells, more selective Envs must be employed for the specific targeting of a particular cell type in the midst of others. When possible, specificity can be achieved through the targeting of cellular receptors expressed uniquely on the cell type of interest. This is the case for the C-type lectin-like receptor (DC-SIGN) expressed almost exclusively on primary DCs [125]. An interesting step forward in the specific transduction of DCs has come through the modification of the envelope glycoprotein of the Sindbis virus. In its natural context this envelope is not specific, since it binds DC-SIGN on DCs, but also to heparan sulfate moieties present on most cell types. However, the removal of the heparan sulfate binding domain from the Sindbis virus envelope protein led to a modified glycoprotein that lost its ability to bind heparan sulfate but retained a strong DC-SIGN binding. Thus, this modification effectively restricted the tropism of pseudotyped lentiviruses (LVs) to DCs both *ex vivo* and *in vivo* [126]. Similar strategies may be applied to other cell types and may be particularly useful to reduce side effects due to widespread transgene expression [127].

A second property specified by some envelope proteins is the ability to influence steps that are subsequent to viral entry into target cells. For example, the infection of quiescent B and T cells is inefficient with VSVg-pseudotyped LVs. This is not due to a defect in viral entry, but rather to a restriction at the step of reverse transcription that is likely a consequence of the poor activation status of these cells [128]. Efficient transduction can be achieved upon cell activation, but this may not be an option in applications in which the preservation of a quiescent state is sought. To achieve efficient cell infection in the absence of major activation signals, a number of strategies have been developed that rely on artificially engineered envelopes and on envelopes other than VSVg. A common trait of these strategies is that envelope molecules, displayed at the surface of the viral particle, trigger an activation signal upon engagement of the cellular receptor. This activates the cell transiently and allows an efficient infection. The strategy has been successfully employed in the transduction of quiescent B and T cells using the Measles virus (MV) gp protein and cytokine-displaying LVs, respectively [129,130]. In these cases, the intracellular signals conveyed upon the engagement of the signaling lymphocytic activation molecule receptor (SLAM) or of the specific cytokine receptor seem sufficient to promote LVs infection, at least transiently. A number of other viral particle-delivered signals are being explored and in the future we can expect a number of interesting developments along these lines.

Finally, certain envelope proteins determine a peculiar intracellular behavior of VNCs that is particularly interesting, as in the case of the rabies G protein that endows pseudotyped particles with the same properties of the rabies virus, namely the ability to undergo retrograde transport along the cell’s axons. This extraordinary property has been used for the transduction of motor neurons in an animal study for the amyotrophic lateral sclerosis (ALS), a progressive neurodegenerative disease. In this study, EIAV-derived lentiviral vectors were pseudotyped with the rabies G protein and injected in limb muscles of mice [131] where, thanks to their envelope, they were able to transduce motor neurons of the central nervous system [132]. The possibility of taking advantage of retrograde transport to achieve neuron transduction from the periphery is extremely attractive for gene therapy applications of the CNS, since this strategy is less invasive than the direct modification of the CNS.

In conclusion, envelope proteins not only specify the type of cells that are recognized by the viral particle, but can also influence the subsequent behavior of the virus inside the cell. In some instances, this contributes to remove some of the post-entry obstacles that can be encountered during the transduction of a particular cell type.

### 3.2. Manipulation of Viral Non-Structural Proteins to Promote Cell Type Specific Targeting: The Case of the SIV_SM_/HIV-2 Vpx Protein and Myeloid Cells

A further possibility to act on the specificity of LV transduction may come from the use of viral proteins that affect the early phases of infection in a cell type dependent manner. One example in this direction is provided by the Vpx protein. Vpx is coded by members of the SIV_SM_/HIV-2 lineage, but is absent in most of the remaining lineages of primate lentiviruses. Vpx is a nonstructural viral protein that is incorporated into viral particles and is thus present during the early phases of infection, where it exerts a positive effect on the process of reverse transcription [133–137]. An interesting feature of the action of Vpx is its cell type specificity that is restricted to cells of the myeloid lineage. The exact mechanism underlying the positive effect of Vpx on lentiviral transduction is currently under investigation. However, multiple lines of evidence suggest that Vpx may act by counteracting a restriction factor specifically expressed in myeloid cells, thus explaining its cell type specific effect [134,138]. Vpx displays two phenotypes that may be of interest for gene therapy purposes. First, it is absolutely required for the infection of myeloid cells with parental SIV_SM_ or HIV-2 viruses, while it is dispensable for the infection of other cell types, such as lymphocytes [139]. Second, when provided onto recipient cells at the moment of infection, it increases the efficiency of transduction of heterologous lentiviral vectors (as HIV-1 and FIV) by at least ten-fold, an effect that is again specific to myeloid cells [133,140]. As a consequence, one can imagine that SIV_SM_ or HIV-2 vectors devoid of Vpx could be used to transduce preferentially peripheral blood lymphocytes in the blood as opposed to monocytes or circulating DCs, thus diminishing anti vector/transgene responses mediated by these antigen presenting cells. Conversely, in applications in which specific transduction of myeloid cells is required, lentiviral vectors (derived from HIV-1, but also FIV and EIAV) may be used together with Vpx, since this will improve their performance specifically in these cells. As a consequence, efficient cell transduction can be achieved even at low viral inputs [133,140], thus avoiding large viral doses that in some instances have been shown to be detrimental to the physiology of modified cells [141,142]. For the moment, Vpx constitutes the sole example of non-structural viral protein that affects, to such a large extent, the efficiency of the early phases of infection in a setup of interest for gene therapy. It is possible that other lentiviral proteins exert other interesting effects that could be similarly exploited, but whether this is the case remains unknown at present. In the future, combinations between specific envelope proteins, viral proteins that have cell type specific properties, and the use of cell type specific promoters, may be particularly potent in promoting the selectivity of the gene transfer process.

## 4. Particular Considerations on the Use of Lentiviral Vectors

### 4.1. The Problem of Cross-Species Usage of Lentiviral Vectors: The Tripartite Motif 5α Protein

Cells possess several intrinsic defense mechanisms against pathogens. The key determinant for these defenses is the recognition of features that are shared by classes of pathogens, or pathogen associated molecular patterns (PAMPs). For retroviruses, two key features are the reverse transcription process and viral capsids that can be truly defined as PAMPs. These are recognized by two prototypical antiviral factors: by members of the APOBEC3 family and by the Tripartite motif 5α protein (TRIM5α), respectively [143,144]. For gene therapy purposes, the classical antiviral effect of APOBEC3s may be neglected using virus-producing cells in which these proteins are absent, as 293T cells. However, the same is not true for TRIM5α that recognizes incoming viral particles in target cells [145–147]. TRIM5α proteins recognize incoming VNCs via CA, triggering their premature disassembly and thus impairing reverse transcription and infection [148]. It is unclear whether this process involves direct degradation of viral components and/or trafficking of VNCs, in particular intracellular locations [149,150], but the result of this impairment can be particularly strong (from 2–3 to 100 fold in a single round infection) (reviewed in [151]). As a general rule, viruses that thrive in one species are not recognized by the TRIM5α ortholog of that species. This is intuitive; to succeed infection a virus must have evolved to bypass this block. On the other hand, TRIM5α orthologs act as a potent barrier in cross-species transmission and can antagonize viruses, and thus vectors, of different species as summarized in Table I [152–157]. Thus, the TRIM5α effect must be carefully weighted when using LVs derived from different species, considering that even a moderate inhibition by TRIM5α may considerably reduce the proportion of successfully transduced cells. One of the features of the restriction mediated by TRIM5α is the fact that it can be saturated with high viral inputs. Generally, saturation is observed within the high viral doses used in gene therapy, suggesting that infection could be achieved with LVs of different species even in the presence of a restricting TRIM5α. However in this case, the need to saturate TRIM5 with high viral inputs may contrast with the requirement to use low viral doses to minimize modifications of the cell physiology or to diminish immune responses directed against the vector [141,142]. In cells exquisitely susceptible to the presence of pathogens, such as myeloid cells, a correct balancing between the requirement for efficient cell modification and the need to avoid gross modifications of the cell physiology may be particularly important.

### 4.2. The Yin and the Yang of Integration

Although the main interest of retroviral vectors for gene therapy purposes lays in the integration of their genome, this very feature is intrinsically dangerous. The potential drawbacks of integration have not received much attention in the past, probably because no adverse effects had been observed in retroviral-mediated gene therapy trials started more than two decades ago (as, for example, in the severe combined immunodeficiency syndrome due to a deficiency in the adenosine deaminase enzyme, SCID-ADA, [183]). However, serious side effects were observed in a proportion of SCID-γC patients undergoing retroviral mediated gene replacement. The SCID-γC syndrome is a lethal immunodeficiency in which T cells cannot develop due to the absence of the γC gene and the gene therapy approach in this trial consisted of a simple gene replacement in hematopoietic stem cells. The results of this clinical trial best illustrate the benefits and the potential dangers linked to retroviral mediated gene therapy. On one hand, the gene replacement trial was a success because modified hematopoietic stem cells functionally replenished the patients’ immune system and about half of the patients maintained the use of immune functions for over 10 years. On the other hand, a proportion of patients developed leukemia as a consequence of the deregulated expression of proto-oncogenes adjacent to the retroviral vector integration sites [184].

This finding spurred a series of studies on the features that govern retroviral integration on a genome wide scale. These studies revealed that retroviral integrases display strong differences in the selection of chromosomal integration sites (in active transcriptional units for HIV-1 and close to transcriptional start sites for MLV) [185,186]. In the case of HIV-1, a further bias toward regions containing recognition sequences for cellular co-factors that associate to IN, such as the lens epithelial growth factor p75, (LEDGF) has also been described [187]. LEDGF is a cellular DNA binding factor that associates to HIV-1 IN and that is required for viral DNA integration [188,189]. It is thus not surprising that HIV-1 integration favors sites in which LEDGF is enriched.

Overall, these studies highlighted the fact that in its natural configuration, the integration process is largely stochastic and that the result of integration may just as likely be with or without consequences. This is shown by the different outcomes observed in patients participating in the same clinical trial, as well as by the findings that particular chromosomal features are preferentially, but not exclusively, selected during integration [185,186].

To more selectively target proviral DNA integration, a number of efforts are now being directed either at engineering modified IN fused to well characterized heterologous DNA binding domains or at modifying IN-associated cellular co-factors [190–192]. The efforts to achieve site directed retroviral integration, or chosen integration, are at their infancy and for the moment the results obtained are rather disappointing because of the relative abundance of similar DNA binding sites spread over the genome. To date, a single virus has been shown to be capable of site specific integration: the adeno-associated virus (AAV) that integrates a small fraction of its genomes into the human chromosome 19q13.42 (although more recent data seems to question this specificity, [193,194]. The specificity of this integration is mediated by the Rep 78/68 proteins that recognize a sequence called AAVS1 that is present both on the viral genome and in this region of the human genome. Future strategies aimed at directing retroviral integration may possibly take advantage of the mechanism provided by this virus and transpose it to lentiviruses. Alternatively, site specific integration may be achieved by engineering IN fusion proteins to meganucleases; DNA nucleases that have the peculiarity to recognize long, and thus rarer, DNA binding sequences that can be theoretically present only once in the human genome [195,196]. Whether these approaches are feasible remains to be determined.

### 4.3. Non-Integrative Lentiviral Vectors

An alternative strategy to circumvent the negative effects of integration, is represented by non-integrative LVs that have been developed by a number of laboratories [197–200]. These are vectors bearing inactivating mutations in IN that inhibit the integration of viral DNA [198,201,202]. During the early phases of infection, viral DNA can either integrate into the host genome or exist in the form of episomal DNA bearing one or two long terminal repeats (the so called 1 and 2LTRs circles). These episomes originate in the nucleus upon viral ends joining by cellular ligases (2LTRs), or through the recombination of the two identical ends (1LTRs). Both forms are competent for gene expression in their natural context (for example during wild type HIV-1 infection) [203], as well as in the context of lentiviral vectors. However, due to their lack of origin of replication, these episomes are gradually lost during cell division. On the contrary, viral DNA episomes are stable in non-dividing cells, the preferred targets for lentiviral mediated transduction. Thus, non-integrative LVs could provide a safe setting in gene delivery at least in non-dividing or differentiated cells.

## 5. Conclusions and Perspectives

Lentiviral vectors bears an obvious advantage over other retroviral vectors in that they offer the possibility to efficiently target non-dividing and differentiated cells, such as DCs or neurons. As such, these vectors are of extreme interest for a multitude of gene therapy applications. Although the basics of retroviral and lentiviral vectors are now firmly established, specific applications require careful tailoring of several elements to ameliorate the efficiency of gene transfer. Indeed, the transduction of non-dividing cells cannot be resumed to the mere phase of nuclear import, and several additional obstacles are encountered by viral vectors in a number of differentiated and quiescent cell types. In some instances, these barriers can be overcome through the use of envelope proteins conferring particular properties to the vector or that transiently stimulate the target cells. In others, the same goal can be achieved through the use of viral elements that facilitate transduction of the particular cell type of interest.

Paradoxically, the use of retroviral vectors is hindered by the same process that makes them interesting for gene therapy, *i.e.*, integration. This process is largely nonspecific and, as it has been shown *in vivo*, may either be of no consequence to the cell or lead to serious drawbacks. Although this problem may in theory be minimized in gene therapy applications targeting terminally differentiated cells, the problem of integration is serious. To this end, a number of alternative strategies have been developed, ranging from the redirection of retroviral integration to particular chromosomal locations, to the ablation of the integration process altogether. Although in its infancy, the efforts to redirect retroviral integration must be pursued and researchers may possibly transpose to lentiviruses a mechanism of specific integration used by other viruses. As in the past, all the ameliorations of lentiviral vectors will be the fruitful transposition of basic research discoveries in virology to the field of gene therapy.

## Figures and Tables

**Table 1.** Lentiviruses and lentiviral-derived vectors.

**Virus**	**Host**	**Disease**	**Vector Generation**			**Species Specific Restriction**
			**1^st^**	**2^nd^**	**3^rd^**	

**HIV-1**	Human	Immunodeficiency	[75,120]	[72,73,116]	[115]	OWM, Rabbit, Cow
				
**HIV-2**	Human	Immunodeficiency*	[158–164]	[165]		OWM, Rabbit, Cow
				
**SIV_SM_**	Monkey	Immunodeficiency /-	[166,167]	[166,168]	[168]	NWM, Cow
				
**SIV_AGM_**	Monkey	Immunodeficiency /-	[169]			NWM
				
**EIAV**	Horses	Infectious anemia	[170,171]	[172]	[172]	OWM, Human, Rabbit, Cow
				
**FIV**	Cats	Immunodeficiency	[173]	[174]	[174,175]	OWM, Human, Rabbit, Cow
				
**VNV**	Sheep	Pneumo-encephalitis		[176]		NT
				
**CAEV**	Goat	Arthritis-encephalitis	[177]		[178]	NT
				
**BIV**	Cow	-/acute febrile illness^#^	[179,180]	[181]	[181]	NT

*: HIV-2 is capable of inducing an immunodeficiency that mirrors the one induced by HIV-1, albeit with a prolonged delay from the initial infection.

/-: SIVs do not cause disease in their natural host, but do so in monkeys of different species.

#: BIV strains isolated from taurine cattle (*Bos Taurus*) are mostly apathogenic, while those isolated from Bali cattle (named also Jembrana disease virus, JDV, in *Bos javanicus*) induce an acute febrile illness.

Vectors have been derived from both. The main references are provided for the different packaging vector generations. NWM and OWM: new and old world monkeys. For simplicity, restriction has been ascribed to one or the other group, although certain species within each group may behave differently. NT: not tested.
